# Ocular Involvement in Hereditary Transthyretin Amyloidosis: A Case Series Describing Novel Potential Biomarkers

**DOI:** 10.3390/genes12060927

**Published:** 2021-06-18

**Authors:** Angelo Maria Minnella, Roberta Rissotto, Martina Maceroni, Angela Romano, Romina Fasciani, Marco Luigetti, Mario Sabatelli, Stanislao Rizzo, Benedetto Falsini

**Affiliations:** 1 Institute of Ophthalmology, Università Cattolica del Sacro Cuore, 00168 Rome, Italy; angelomaria.minnella@unicatt.it (A.M.M.); roberta.rissotto@gmail.com (R.R.); stanislao.rizzo@unicatt.it (S.R.); benedetto.falsini@unicatt.it (B.F.); 2 Fondazione Policlinico Universitario A. Gemelli-IRCCS, 00168 Rome, Italy; romina.fasciani@policlinicogemelli.it (R.F.); mluigetti@gmail.com (M.L.); 3 Institute of Neurology, Università Cattolica del Sacro Cuore, 00168 Rome, Italy; angela.romano12@gmail.com (A.R.); mario.sabatelli@unicatt.it (M.S.); 4 Centro Clinico NEMO Adulti, Sede di Roma, 00168 Rome, Italy

**Keywords:** hereditary transthyretin amyloidosis (hATTR), transthyretin (TTR), corneal confocal microscopy (CCM), electroretinogram (ERG), Optical Coherence Tomography (OCT), ocular biomarkers, personalized medicine

## Abstract

Hereditary transthyretin amyloidosis (hATTR) is a rare disease caused by a point mutation in the transthyretin (TTR) gene and inherited in an autosomal dominant fashion. TTR is a plasma protein that functions as a carrier for thyroxine (T4) and retinol (vitamin A). Ophthalmological manifestations are due to both the hepatic and ocular production of mutated TTR. In this case series, we report the ocular manifestations of hATTR in eighteen eyes of nine consecutive patients. Corneal nerve abnormalities as well as morphological and functional changes in the retina were investigated. The study was a single-center, retrospective, observational, clinical case series. In all patients, corneal confocal microscopy (CCM), multimodal imaging of the retina, including fundus photography and Optical Coherence Tomography (OCT), as well as rod and cone electroretinography (ERG) were performed. Eight patients had active disease and one was an unaffected carrier. In all study eyes, corneal nerve plexa examined with CCM were poorly represented or absent. Mixed rod-cone and cone ERG b-wave amplitudes were reduced, and photopic b-wave responses were significantly delayed. Photopic Negative Response (PhNR) amplitude was significantly reduced, while PhNR latency was significantly augmented. In 13/18 eyes, vitreous opacities and abnormalities of vitreo-retinal interface were found. The current results highlight the presence of corneal nerve damage. Functional retinal abnormalities, detected by ERG, can be found even in the presence of minimal or absent structural retinal damage. These findings support the use of CCM and ERGs to detect early biomarkers for primary hATTR.

## 1. Introduction

Hereditary transthyretin amyloidosis (hATTR) [OMIM **#**105210] is a rare disease caused by a point mutation in the transthyretin (TTR) gene and inherited as an autosomal dominant trait with variable penetrance [1,2].

Clinically, it is a severe, heterogeneous, systemic condition caused by the extracellular deposition of amyloid fibrils in various organs and tissues. The deposition of insoluble amyloid fibrils leads to progressive multiorgan dysfunction, with an expected survival of approximately 10 years from onset [1]. More than 130 causative single amino acid substitutions have been discovered, with V30M (p.Val50Met) being the most frequent worldwide [3].

TTR is a plasma protein that functions as a carrier for thyroxine (T4) and retinol (vitamin A) [4]. The major organ responsible for TTR synthesis and secretion into blood circulation is the liver, although it can also be produced by the brain choroid plexuses and by the retinal pigment epithelium (RPE) [1,2,5]. The pathological mechanism leading to systemic symptoms is the disassembly of TTR tetrameric structure into monomers that are prone to precipitation into tissues, where they aggregate into fibrils and cause damage by interfering with the organ function [1,2].

The main organs involved in hATTR amyloidosis are peripheral nerves and heart, resulting in sensorimotor neuropathy, autonomic dysfunction, and cardiomyopathy. However, many other organs, such as the kidney and the eye, can be involved, leading to a severe, multisystem disease with a great variability in clinical presentation and course.

The phenotype, severity, and age of onset may vary depending on the causal pathogenic variant and the geographic area. In endemic areas, hATTR is typically characterized by a high penetrance rate and onset before the age of 50 years (so called early-onset disease, usually presenting in the third to fourth decade) [1,6]. By contrast, outside these regions, a low penetrance rate is common, and patients show a late-onset disease, usually after the age of 60. Furthermore, a positive family history is frequent in endemic areas, reaching up to 98% in Portugal [5], thus facilitating the diagnosis of hATTR. Diagnosis is, by far, more challenging and often delayed by several years in non-endemic areas because of the lack of a family history in 50% to nearly 80% of cases (sporadic presentations) [7].

The pattern of the neurological impairment may vary according to the geographic areas. In the endemic areas, patients presenting with an early-onset hATTR deteriorate quickly because of autonomic dysfunction and rapid progression of the sensory-motor deficit [1]. Conversely, in non-endemic areas many patients present with a late onset hATTR and the polyneuropathy (affecting predominantly the large nerve fibres) progresses slowly, often with cardiac involvement but with less autonomic dysfunction [8,9].

Ophthalmological manifestations are due to both the hepatic and local (ocular) production of mutated TTR [9,10,11,12]; for instance, vitreous opacities are attributable to RPE secretion of the variant protein into the vitreous gel while vascular conjunctival abnormalities result from the liver synthesis of mutated TTR. This has been confirmed by studies on patients affected by hATTR who underwent liver transplantation. It was noted that vitreous deposits of amyloid were not halted after the surgery, but rather worsened [12,13], while vascular conjunctival abnormalities did not progress.

Given the involvement of TTR in the vitamin A retinal metabolism and visual cycle, it is conceivable to hypothesize that in TTR-related amyloidosis, retinal function abnormalities can be present at an early stage of the disease.

To our knowledge, most studies that have been conducted on ocular hATTR only described the clinical stage of the ophthalmic disease. In addition, no functional studies in hATTR have been conducted so far.

The aim of the present study was to investigate potential ocular biomarkers in patients with confirmed hATTR, by evaluating the abnormalities of the corneal nerves as well as the morphological and functional alterations of the retina.

## 2. Materials and Methods

This retrospective study was conducted at the outpatient eye clinic, Fondazione Policlinico Universitario A. Gemelli-IRCCS, Rome (Italy), in accordance with the principles of the Declaration of Helsinki. This research was approved by an internal ethical committee of the same institution and informed consent was obtained from all patients, after a full and detailed explanation of the goals and procedures of the study were provided (ethic code number: ID1493).

### 2.1. Subjects

All patients were selected from a larger cohort that was followed by the Department of Neurology of the same institution. Recruitment was performed according to a collaboration protocol between the two departments and following an internal procedure of ocular evaluation of hATTR patients.

Eighteen eyes of nine patients with a confirmed diagnosis of hATTR were enrolled between March 2019 and July 2019.

Inclusion criteria were: (1) established diagnosis of hATTR confirmed by a positive genetic testing associated with signs or symptoms of the disease and (2) age > 18 years.

The exclusion criteria were: (1) the comorbidity of diabetes, atherosclerotic vasculopathy, glaucoma, or any other macular or retinal disorders, (2) optical media opacity precluding reliable retinal functional exams, and (3) an inability of the patients to maintain visual fixation.

### 2.2. Data Acquisition

Each enrolled patient underwent a global evaluation, with an assessment of disease severity using different scales, including FAP stage, Neuropathy Impairment Score (NIS), and Kumamoto score.

All patients underwent a full ophthalmologic examination, including best corrected visual acuity (BCVA) using Early Treatment Diabetic Retinopathy Study (ETDRS) charts and intraocular pressure (IOP) measurements, as well as anterior segment slit lamp biomicroscopy and indirect fundus ophthalmoscopy. Color fundus photos were taken with Eidon (Centervue, Fremont, CA, USA), while corneal confocal microscopy (CCM) was obtained with Confoscan 4 (Nidek, Gamagori, Japan).

### 2.3. OCT Assessment

Infrared Imaging (IR), Fundus Blue Autofluorescence (BAF) were taken with Heidelberg Spectralis (Heidelberg Engineering, Heidelberg Germany). SD-OCT was performed using Zeiss Cirrus 5000-HD-OCT Angioplex, sw version 10.0, (CarlZeiss, Meditec, Inc., Dublin, CA, USA). A High-definition 5 Line Raster and a macular map (6 × 6 mm Macular Cube 512 × 128) were acquired. OCT qualitative assessment was performed by two independent masked investigators (A.M.M. and M.M.) who assigned a score from 0 to 5 to each considered entity: vitreous, vitreo-retinal interface, and outer retina (normal findings corresponded to a value of 0, qualitative alterations corresponded to a value from 1 to 5). Central Macular Thickness (CMT) was automatically measured using Macular Cube scans. Subfoveal Choroidal thickness (SFCT) and Outer Nuclear Layer (ONL) thickness were manually measured on horizontal OCT B-scans. ONL thickness was manually measured at 5 points from the posterior edge of the Outer Plexiform Layer (OPL) to the External Limiting Membrane (ELM) at the fovea, and at 1.500-micron and 3000-micron intervals temporal and nasal to the fovea. An average thickness for ONL was calculated from the 5 values obtained.

Twenty-six eyes of 13 healthy patients were evaluated as controls for OCT measurements.

### 2.4. Electroretinogram Assessment

Ganzfeld mixed rod-cone (dark adapted) and cone-mediated (light adapted) electroretinograms (ERGs) were recorded in all patients according to a previously published shortened protocol [14]. Briefly, all ERGs were recorded while patients fixated monocularly a 0.258 central fixation mark with pupils pharmacologically (1%tropicamide and 2.5% phenylephrine hydrochloride) dilated to a diameter ‡ 8 mm. Following a dark-adaptation period of 30 min, the Ganzfeld mixed rod-cone ERG was recorded in response to white 50 microsec flashes of 1 cd_s/m^2^. The responses were averaged over 20 stimulus presentations. Interstimulus interval was 10 s. Following a 20-min adaptation to light, Ganzfeld cone mediated ERG was recorded in response to white 50-microsec Ganzfeld stimuli with an intensity of 2 cd_s/m^2^ presented on a steady white background of 20 cd/m^2^ of a Ganzfeld bowl. Responses were averaged over 40 stimulus presentations. Interstimulus interval was 1 s. Signals were amplified (50 K), filtered (0.3–250 Hz), digitized at 2 KHz, and averaged over 40 runs with automatic artifact rejection. The a- to b-wave amplitudes, as well as the b wave implicit time were measured for both rod-cone and cone ERGs. Photoreceptor responses from a-waves were not measured. For each patient, the Photopic Negative Responce (PhNR) from the single flash cone-mediated responses [14,15,16] was measured in its amplitude and peak time The instrument used was Retimax, (CSO Company, Florence, Italy). A group of 40 healthy eyes with no signs of any ocular disease served as controls.

### 2.5. Statistical Analyses

The percentage distribution by gender and qualitative parameters was calculated; measures of central tendencies (mean, range) were obtained for quantitative parameters. The results were analyzed by parametric tests (*t*-test and analysis of variance) assuming normal distribution.

In all the analyses, a *p* < 0.05 was considered as statistically significant.

## 3. Results

### 3.1. Demographic, Genetic and Systemic Findings

This case series involved a cohort of six males and three females, aged between 51 and 87 (mean 66.8), and with age at diagnosis between 48 and 81 (mean 63.7). The mean disease duration was 35.42 months. Two patients, a male and a female, were siblings and became symptomatic during carrier monitoring. Only one woman was an asymptomatic carrier, while all the other patients disclosed symptoms of the disease. The only examined carrier reported sensory symptoms in lower limbs but neurophysiological investigations (nerve conduction studies and Sudoscan) were unremarkable, as well as cardiological tests; therefore, we studied the eyes in order to detect possible ophthalmological signs of disease onset. TTR gene sequencing revealed V30M (p. Val50Met) pathogenic variant in three patients, F64L (p. Phe84Leu) in three patients (two siblings), A109S (p. Ala129Ser), V122I (p. Val142Ile) and T59K (p. Tyr79Lys) in one patient each [17].

None of the patients underwent hepatic transplant, therefore, it was not possible to assess whether ocular manifestations might be affected by this surgical procedure, as suggested by some studies in the literature.

Polyneuropathy was confirmed by electromyography/nerve conduction studies in all affected patients (except for the asymptomatic carrier), while cardiomyopathy was found in five out of nine (56%) patients. Gastrointestinal involvement, defined as malabsorption, constipation, or diarrhea, was present in four patients (45%).

With regards to systemic therapy, all affected patients were taking specific drugs for hATTR: three patients were assuming Tafamidis as a single therapy, two patients Patisiran in monotherapy, two patients a dual therapy with Tafamidis and Inotersen and Tafamidis and Patisiran respectively, and the remaining one Diflunisal.

The genetic, demographic, and clinical data are reported in detail for each patient in Table 1. 

### 3.2. Ocular Findings

All data concerning ophthalmological examination are reported in detail in Table 2.

(a) Best Corrected Visual Acuity

Best corrected visual acuity (BCVA) was substantially preserved in all subjects, except in patient #1 who had a posterior capsule opacification in right eye and outer retinal abnormality in left eye. A mean BCVA of 84 ETDRS letters (range 56–90) was found.

(b) Anterior Segment

Corneal alterations found in patients included absent or poorly represented subepithelial nervous plexus, with abnormal nerve extension and density, together with nerve segmentation and fragmentation, accompanied by thinning of stromal nerves, generally with a normal keratinocyte density. These alterations characterized all the eighteen examined eyes, with variable severity. Corneal confocal microscopy (CCM) findings are reported in Table 3. Interestingly, the only examined carrier (pt #5 in Table 1 and Table 3) had abnormalities detected with CCM, namely thinned stromal nerves in both eyes, despite neurophysiological investigations (nerve conduction studies and Sudoscan) being unremarkable, as well as cardiological tests.

The intraocular pressure (IOP) was within normal limits in all subjects, with a mean IOP of 13 mmHg. None of the examined patients had a diagnosis of glaucoma.

Five eyes (27.8%) were pseudophakic and two of them presented a posterior capsule opacification. Four eyes (22.2%) showed lens opacities, albeit the BCVA was not affected (see Table 2).

(c) Posterior Segment

On ophthalmoscopic examination, none of the examined patients showed evident vitreous opacities. Regarding retinal OCT assessment, 26 normal control eyes of 13 healthy patients (5 males, 7 females) were evaluated for comparison. The OCT findings are reported in detail in Table 2. In the hATTR group, qualitative assessment of OCT structural images revealed vitreal abnormalities in 13 out of 18 eyes (72%), vitreo-retinal interface alteration in 13 out of 18 eyes (72%), and abnormalities of the outer retinal layers in 4 out of 18 eyes (22%). CMT and SFCT were slightly reduced in hATTR patients (257.33 ± 24.46 μm and 272.22 ± 60.68 μm) in comparison to normal controls (264.57 ± 13.36 μm and 278.80 ± 37.92 μm), albeit the result was not statistically significant.

A statistically significant difference was found for ONL thickness (*p* = 0.002) between hATTR patients and control group (72.57 ± 8 μm vs. and 79.5 ± 6.05 μm, respectively).

(d) Functional Studies

Mixed ERG B wave amplitude was below the lower 95% normal confidence limit (below 2 SDs range compared to reference values) in 33.3% of the eyes, while photopic ERG B wave amplitude resulted below 2 SDs range from the reference values in 5.5% of eyes. Sixteen (88.8%) examined eyes had a delay in photopic B wave peak time, above 33 ms + 2 SDs (upper 95% normal confidence limit). Photopic Negative Response (PhNR) amplitude was abnormal in fifteen eyes (83.3%), below 9.17 μV (19.17 μV–10 μV), whereas PhNR peak time resulted abnormal in sixteen patients (88.8%), above the upper 95% normal confidence limit of 48 ms (Table 4).

Our data showed that the amplitude of responses was greatly reduced compared to reference values (2 SDs range), with abnormalities in mixed rod-cone (*p* < 0.0001) and cone (*p* = 0.2, ns) ERG b-wave amplitudes. Photopic b-wave responses were significantly delayed in comparison to normal reference values (*p* < 0.0001).

Furthermore, in hATTR patients, PhNR amplitude was significantly reduced (*p* < 0.0001), while PhNR latency was significantly augmented (*p* < 0.0001) compared to normal controls (Table 5).

No significant correlation was found between ERG amplitudes and OCT parameters. ERG peak time tended to increase as the thickness of subfoveal choroidal bed decreased.

**Figure 1:**
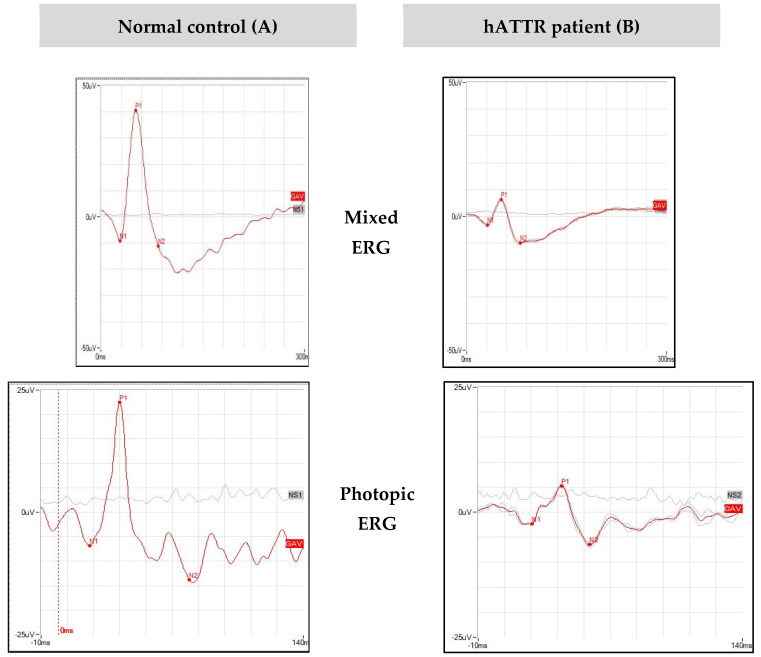

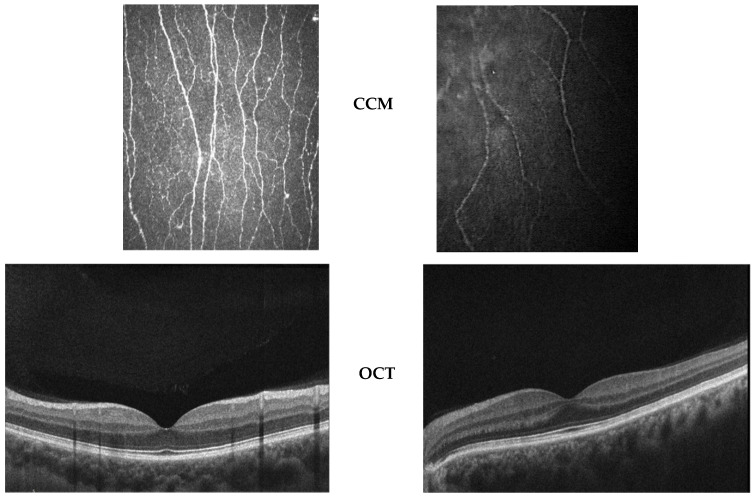
ERGs, CCM, and OCT of a healthy patient vs. an hATTR patient. Top to bottom: mixed Electroretinogram (ERG), photopic ERG, Corneal Confocal Microscopy (CCM), OCT B scan of a normal 43 year old control (**A**) and a 54 years old hATTR patient (**B**) RP. (**A**) shows normal ERG parameters, as well as normal nervous corneal plexus. OCT scan reveals a preserved morphology and reflectivity of all retinal layers, with a mean ONL thickness of 84 microns. (**B**) shows reduced ERG b-wave amplitudes and a poorly represented corneal nervous plexus. No alterations of retinal morphology and reflectivity are observed on OCT scan. Mean ONL thickness is reduced in comparison to normal control (61 microns).

Figure 1A shows normal ERG parameters, as well as normal nervous corneal plexus. OCT scan reveals a preserved morphology and reflectivity of all retinal layers, with a mean ONL thickness of 84 microns. Figure 1B shows reduced ERG b-wave amplitudes and a poorly represented corneal nervous plexus. No alterations of retinal morphology and reflectivity are observed on OCT scan. Mean ONL thickness is reduced in comparison to normal control (61 microns).

## 4. Discussion

The present study was designed to evaluate the ocular findings of nine patients affected by hATTR amyloidosis. In particular, the main aim of the study was to evaluate morphological and functional parameters of the cornea and retina in primary hATTR patients, in the attempt to identify potential biomarkers of a pre-symptomatic stage of ocular involvement.

We examined patients with four different pathogenetic variants, all described in Italy: V30M (p. Val50Met), F64L (p. Phe84Leu), A109S (p. Ala129Ser), V122I (p. Val142Ile), and T59K (p. Tyr79Lys) [18]. V30M (p. Val50Met) and F64L (p. Phe84Leu) are the two most frequent mutation in Lazio, our region from central Italy, as recently described [19].

The main finding of this study was that the large majority of patients showed significant abnormalities of scotopic and photopic ERGs, including the PhNR, the negative potential following the b-wave, representing the activity of Retinal Ganglion Cells (RGCs) and their axons [20]. The ERG abnormalities, which were detected in all patients, may be related to a malfunction of TTR protein leading to a perturbation of visual cycle resulting in a reduced supply of retinoids, for both rod and cone photoreceptors. In this respect, it will be important in the future to explore the retinal function recovery after bleaching, as a process mediated by the visual cycle.

Concerning structural ocular involvement, OCT images revealed vitreous opacity and structural retinal abnormalities in hATTR patients. Although vitreous opacities are considered characteristics of ocular hATTR, the OCT structural abnormalities detected in our study are a novel finding and are likely to be related to the systemic disease. Indeed, a loss of ONL could be a consequence of a reduced retinoid supply and a reduced number of photoreceptors. Similarly, the thinning of subfoveal choroidal bed at least in some study eyes can be related to a photoreceptor loss associated to ONL thinning. A similar effect can be observed in inherited retinal degenerations [21].

CCM is currently the only non-invasive technique useful for detecting corneal alterations and early nerve damage in vivo. This technique was used in our study to show corneal pathologic changes (see Table 3). CCM was shown to be abnormal in all the eighteen examined eyes, revealing an absent or poorly represented subepithelial nervous plexus and thinner stromal nerves. It is worth noting that an overt damage to corneal stromal and subepithelial plexus was found in the carrier patient (patient #5) who did not show any impairment in heart and peripheral nerves. Further studies are needed to support the use of CCM as a technique for early detection of systemic neuropathy in asymptomatic or carrier patients, even when other systemic instrumental examinations result as negative. As shown by some authors [22,23], the paucity of nerves in the different layers of the cornea, detected with CCM, may be used as a surrogate marker of denervation in diabetic sensorimotor and autonomic neuropathy. Rousseau et al. investigated whether CCM may correlate with the severity of polyneuropathy in hereditary ATTR amyloidosis patients. This work showed that corneal nerve fiber length correlated with the severity of both sensorimotor and autonomic neuropathies in hATTR, as well as with clinical motor neuropathy and walking status and with IntraEpidermal Nerve Fiber Density (IENFD) in the lower limbs [24]. Rousseau and colleagues [24] highlighted that corneal nerve fiber length (CNFL) can be thinner in patients with hereditary hATTR amyloidosis compared to matched healthy controls, and that it correlates negatively with the severity of the neuropathy. Consequently, CNFL as measured by confocal microscopy could be employed as a useful and sensitive marker of denervation in hATTR amyloidosis, showing many diagnostic and clinical advantages compared to preexisting methods [24]. Further studies are needed to investigate whether CCM could be employed as a reproducible method of CNFL measurement and whether it could be useful to monitor amyloidosis disease progression in patients receiving novel therapies for amyloidosis.

## 5. Conclusions

Our data suggest that a complete ophthalmological examination is useful in hATTR patients to detect a subclinical ocular involvement. The main findings of the study are the alterations of retinal function, even in the presence of minimal or absent structural retinal damage, in association with corneal nerves abnormalities, in patients with a confirmed diagnosis of primary hATTR amyloidosis and no visual impairment.

CCM and electrophysiological tests revealed bilateral involvement in all patients. Sub-epithelial nervous plexus alterations and thinning of stromal nerves, as well as ERG abnormalities might be used as specific early biomarkers for peripheral denervation, both in patients with overt disease and in carriers. CCM and ERG are quick, non-invasive, and easily applicable examinations and are potentially useful as screening tests for hATTR patients.

## Figures and Tables

**Table 1:** Genetic, demographic, and clinical data of the participants.

Patient	Sex	Age	Age of Onset	Age at Diagnosis	Follow-Up (Months)	TTR Pathogenic Variant	FAP Stage	NIS (0–244)	Kumamoto sco–re (0–120)	Inheritance	Systemic invo–lvement			Treatment (Months Since Starting)
											PN	CM	GI	
**#1**	F	87	79	81	84	V30M	III	140.25	30	U	Yes	Yes	Yes	**Diflunisal (12)**
**#2**	M	64	58	59	76	V30M	II	73.75	30	U	Yes	Yes	Yes	**Patisiran (48)**
**#3**	M	79	77	79	11	V122I	I	21	36	U	Yes	Yes	No	**Tafamidis (10)**
**#4**	M	76	65	70	19	A109S	II	58.7	28	U	Yes	No	Yes	**Patisiran (16)**
**#5**	F	52	-	-	10	V30M	0	0	0	Maternal	No	No	No	**None**
**#6**	F	58	58	58	19	F64L	I	4	3	Paternal	Yes	No	No	**Tafamidis (8)**
**#7**	M	51	51	51	19	F64L	I	12.50	0	Paternal	Yes	Yes	No	**Tafamidis (8)**
**#8**	M	56	47	48	20	T59K	II	123	35	Maternal	Yes	Yes	Yes	**Tafamidis (77) + Inotersen (7)**
**#9**	M	78	71	71	8	F64L	II	88	28	U	Yes	No	No	**Tafamidis (72) + Patisiran (8)**

Legend: FAP, Familial Amyloid Polyneuropathy; NIS, Neuropathy Impairment Score; PN, polyneuropathy; CM, cardiomyopathy; GI, gastrointestinal; F, female; M, male; U, unknown. In patients #6 and #7, age at diagnosis refers to onset of symptoms during monitoring of carriers status.

**Table 2:** Ocular findings.

Patient	Eye	BCVA (ETDRS Letters)	IOP (mmHg)	Lens Condition	Vitreous Opacities on Ophthalmos Copy	OCT Finding				
						Vitreous Opacity (0–5)	V-R Interface Alterations (0–5)	OR Alterations (0–5)	CMT and SFCT (µ)	ONL (µ)
**#1**	RE	77	18	Pseudofakia, PCO	No	3	4	1	245 and 320	69.6
	LE	56	15	Pseudofakia, PCO	No	3	3	4	290 and 330	52.8
**#2**	RE	90	14	PPC	No	1	1	0	270 and 330	70.4
	LE	87	12	PPC	No	1	1	1	280 and 350	61.8
**#3**	RE	85	11	Normal	No	0	0	0	244 and 180	82.2
	LE	85	13	Normal	No	0	0	0	231 and 160	84.8
**#4**	RE	88	10	CNC	No	3	1	0	279 and 300	79.4
	LE	87	10	CNC	No	2	1	0	287 and 270	74.2
**#5**	RE	90	13	Normal	No	2	1	0	211 and 300	70.6
	LE	90	16	Normal	No	1	0	0	207 and 310	71.8
**#6**	RE	85	15	Normal	No	2	2	0	281 and 330	73.2
	LE	85	16	Normal	No	2	2	0	276 and 244	71.2
**#7**	RE	88	12	Normal	No	0	1	0	262 and 250	75.4
	LE	87	12	Normal	No	0	1	0	266 and 250	77.8
**#8**	RE	88	10	Pseudofakia	No	2	2	0	237 and 248	74
	LE	88	11	Normal	No	1	1	0	237 and 240	82.8
**#9**	RE	86	16	Pseudofakia	No	0	0	1	269 and 238	61.2
	LE	85	16	Pseudofakia	No	1	0	0	260 and 150	73.2

Legend: BCVA: best corrected visual acuity; CMT: central macular thickness; CNC: cortico-nuclear cataract; IOP: intraocular pressure; LE: left eye; ONL: outer nuclear layer; OR: outer retina; PCO: posterior capsule opacification, PPC: posterior polar cataract; RE: right eye; SFCT: sub-foveal choroidal thickness; V-R: vitreo-retinal. A score from 0 to 5 was assigned to each considered entity: vitreous, vitreo-retinal interface, and outer retina (normal findings corresponded to a value of 0, qualitative alterations corresponded to a value from 1 to 5).

**Table 3:** Corneal alterations.

Patients	Eye	CCM			Other Remarks
		Absent/Rarefied Subepithelial NP (Extension and Density)	Nerve Segmentation and/or Fragmentation	Thinning of Stromal Nerves	Deposits between Bowman and Stroma
**#1**	RE	yes	no	no	yes
	LE	yes	no	no	yes
**#2**	RE	yes	no	no	yes
	LE	yes	no	no	yes
**#3**	RE	yes	yes	no	no
	LE	yes	yes	no	no
**#4**	RE	yes	yes	no	no
	LE	no	yes	no	no
**#5**	RE	no	yes	no	no
	LE	no	yes	no	no
**#6**	RE	no	no	yes	no
	LE	no	no	yes	no
**#7**	RE	no	yes	yes	yes
	LE	no	yes	yes	yes
**#8**	RE	yes	yes	no	no
	LE	yes	yes	no	no
**#9**	RE	yes	yes	no	no
	LE	yes	yes	no	no

Legend: CCM: corneal confocal microscopy; LE: left eye; RE: right eye.

**Table 4:** Electroretinogram results.

Patient	Eye	Mixed ERG		Photopic ERG		PhNR	
		B Wave Amplitude (µV)	B Wave Peak Time (ms)	B Wave Amplitude (µV)	B Wave Peak Time (ms)	Amplitude (µV)	Peak Time (ms)
**#1**	RE	53.22	53.91	29.84	37.2 *	7.19 *	55.08 *
	LE	47.47	52.73	33.63	36.62 *	4.35 *	51.86 *
**#2**	RE	25.73	50.39	18.60	33.40 *	7.67 *	51.86 *
	LE	24.76 *	50.39	19.46	33.40 *	5.83 *	50.39 *
**#3**	RE	34.97	60.35 *	15.97	39.84 *	8.43 *	58.59*
	LE	42.33	59.18	17.70	41.31 *	7.04 *	50.98 *
**#4**	RE	14.06 *	50.98	10.67	37.21 *	4.62 *	54.79 *
	LE	9.39 *	51.56	7.63 *	37.21 *	7.03 *	53.32 *
**#5**	RE	23.38	46.29	22.12	32.81	7.66 *	47.75
	LE	23.44 *	46.88	23.00	32.52	9.44	47.17
**#6**	RE	34.45	49.80	29.91	33.40 *	8.12 *	50.10 *
	LE	35.78	49.22	28.06	33.69 *	9.70	50.39*
**#7**	RE	25.46	50.98	22.06	34.57 *	5.94 *	50.39 *
	LE	30.53	52.73	21.83	34.57 *	5.55 *	50.98*
**#8**	RE	30.22	48.63	28.75	34.28 *	7.28 *	49.51 *
	LE	19.03 *	49.22	17.61	34.57 *	6.40 *	50.39 *
**#9**	RE	22.34	50.98	21.83	34.86 *	9.99	73.24*
	LE	24.75 *	52.15	18.70	35.45 *	5.68 *	74.12 *

LE: left eye; RE: right eye; *: altered values.

**Table 5:** Electroretinogram results of hATTR patients vs. controls.

	Mixed ERG				Photopic ERG				PhNR			
	Amplitude (μV)		Peak Time (ms)		Amplitude (μV)		Peak Time (ms)		Amplitude (μV)		Peak Time (ms)	
	RE	LE	RE	LE	RE	LE	RE	LE	RE	LE	RE	LE
**hATTR patients**												
*Mean*	29.31	28.60	51.36	51.56	22.19	20.84	35.28	35.48	7.43	6.78	54.59	53.28
*Std. deviation*	10.40	11.13	3.71	3.25	6.19	6.85	2.18	2.49	1.42	1.68	7.3	7.5
*N of patients*	9				9				9			
**Normal controls**												
*Mean*	50.55	49.28	52	52	25.70	25.76	29	29	19.17	19.17	40	40
*Std. deviation*	16.36	10.86	4	4	9.23	8.35	2	2	5	5	4	4
*N of patients*	40				40				40			

ERG: electroretinogram; LE: left eye; RE: right eye.

## Data Availability

Data available from authors.
